# Case Report: Serplulimab-induced checkpoint inhibitor pneumonitis in extensive-stage small cell lung cancer with underlying COPD: a case report and literature review

**DOI:** 10.3389/fonc.2026.1935484

**Published:** 2026-10-05

**Authors:** Jiangang Ju, Qiang Shen, Qingqing Chen, Linfeng Shen, Xiaofang Fu

**Affiliations:** Department of Respiratory and Critical Care Medicine, Zhejiang University School of Medicine Second Affiliated Hospital Linping Campus, Hangzhou, Zhejiang, China

**Keywords:** case report, checkpoint inhibitor pneumonitis, chronic obstructive pulmonary disease, extensive-stage small cell lung cancer, serplulimab

## Abstract

**Background:**

Serplulimab, a first-line therapy for extensive-stage small cell lung cancer (ES-SCLC), can cause checkpoint inhibitor pneumonitis (CIP). The clinical features and outcomes of serplulimab-induced CIP in patients with Global Initiative for Chronic Obstructive Lung Disease (GOLD) stage IV chronic obstructive pulmonary disease (COPD) remain unclear.

**Case description:**

An 80-year-old man with ES-SCLC and preexisting GOLD stage IV COPD received first-line serplulimab plus chemotherapy. After achieving an initial partial response, chemotherapy was discontinued due to reduced tolerance following bacterial pneumonia, and he was continued on serplulimab monotherapy. Three days after the first maintenance dose of serplulimab, he presented with acute severe chest tightness, dyspnea, and hypoxemia and was admitted to the hospital 4 days later. Chest CT revealed new bilateral pulmonary infiltrates. Extensive workup for infection, acute exacerbation of COPD, and tumor progression was negative; the case was ultimately classified as grade 4 serplulimab-induced CIP. He was treated with high-dose methylprednisolone, intravenous immunoglobulin, and empiric anti-infective therapy, with significant clinical improvement. Follow-up CT at 2 months post-discharge showed significant resolution of pulmonary infiltrates.

**Conclusions:**

Severe, life-threatening CIP can occur in elderly patients with ES-SCLC and preexisting GOLD stage IV COPD. In this patient, prompt recognition, intensive immunosuppression, and permanent programmed death-1 (PD-1) inhibitor discontinuation contributed to a favorable outcome. Whether preexisting GOLD stage IV COPD confers an increased risk of severe CIP in this understudied population warrants further investigation.

## Introduction

Small cell lung cancer (SCLC), the most aggressive and metastatic form of lung cancer, accounts for approximately 10%–15% of all lung cancer cases and contributes to the high global morbidity and mortality rates of lung cancer (1). Serplulimab, a fully human anti-programmed death-1 (anti-PD-1) monoclonal antibody developed in China, has been established as a first-line therapy for extensive-stage SCLC (ES-SCLC) in combination with etoposide and carboplatin (2). With the widespread clinical application of immunotherapy, immune-related adverse events, especially checkpoint inhibitor pneumonitis (CIP), have attracted growing clinical attention. Reported incidences of CIP vary widely (2.6%–33.0%) depending on tumor type, regimen, and baseline pulmonary comorbidities (3–5), and severe cases are associated with increased mortality (6). In first-line chemoimmunotherapy for ES-SCLC, CIP is consistently rare: the phase III ASTRUM-005 trial of serplulimab plus chemotherapy reported all-grade CIP in 0.8% and grade ≥3 CIP in 0.3% of the patients (7); similarly low rates were observed in CASPIAN (durvalumab, 2.1%) (8) and IMpower133 (atezolizumab, 2.3%) (9). Although cross-trial comparisons should be interpreted with caution, selected studies in the recurrent/refractory setting have reported numerically higher rates, particularly with dual checkpoint inhibition (e.g., 3.1%–5.6% in CheckMate 032, including one pneumonitis-related death in the nivolumab 3 mg/kg plus ipilimumab 1 mg/kg cohort) (10). For serplulimab-related CIP, the median time to onset was approximately 101 days, indicating that CIP can occur beyond the initial cycles of treatment (7). Notably, patients with severe pulmonary impairment are typically excluded or underrepresented in such registration trials, including those with preexisting Global Initiative for Chronic Obstructive Lung Disease (GOLD) stage IV chronic obstructive pulmonary disease (COPD).

Although safety data on serplulimab continue to accumulate, and preexisting COPD has been recognized as a potential risk factor for CIP (2, 7, 11–14), specific evidence on serplulimab-induced CIP in patients with GOLD stage IV COPD remains scarce, and clinical evidence to guide practice in this population is limited. Herein, we report an elderly ES-SCLC patient with preexisting GOLD stage IV COPD who developed CIP during serplulimab treatment and achieved marked clinical improvement after immunosuppressive therapy. Additionally, we discuss the clinical manifestations, diagnostic challenges, and management strategies of this immune-related adverse event and complement the case report with a focused literature review summarizing current evidence of CIP in PD-1/PD-L1 inhibitor-treated SCLC so as to provide preliminary clinical insights for the timely identification and management of CIP in high-risk ES-SCLC patients with GOLD stage IV COPD.

## Case presentation

An 80-year-old man, who had a 40-year smoking history (20 cigarettes per day) and GOLD stage IV COPD on triple inhaled maintenance therapy, was diagnosed with extensive-stage SCLC 5 months earlier. Chest CT at that time revealed space-occupying lesions in both the left upper and left lower lobes (**Figure 1A**). Subsequent bronchoscopic biopsy confirmed extensive-stage SCLC (cT4NxM1a), with immunohistochemistry showing CD56 (+), CKpan (+), CgA (−), Ki-67 (95%+), LCA (−), P63 (−), Syn (+), TTF-1 (+), P53 (partially+), and INSM1 (+). PD-L1 expression was not assessed, in accordance with institutional practice at the time, as PD-L1 status does not alter first-line chemoimmunotherapy recommendations for SCLC. His baseline Eastern Cooperative Oncology Group (ECOG) performance status (PS) was 2, and he received first-line carboplatin/etoposide/serplulimab every 21 days. After 5 cycles (cumulative serplulimab dose 1,500 mg), follow-up CT demonstrated a partial response (**Figure 1B**). However, following an episode of left lower lobe bacterial pneumonia, the patient’s performance status deteriorated to ECOG PS 3. After the pneumonia was controlled with antibiotics and following discussion with the family, chemotherapy was discontinued; serplulimab 300 mg was continued as maintenance on 27 September 2025 (sixth dose).

**Figure 1:**
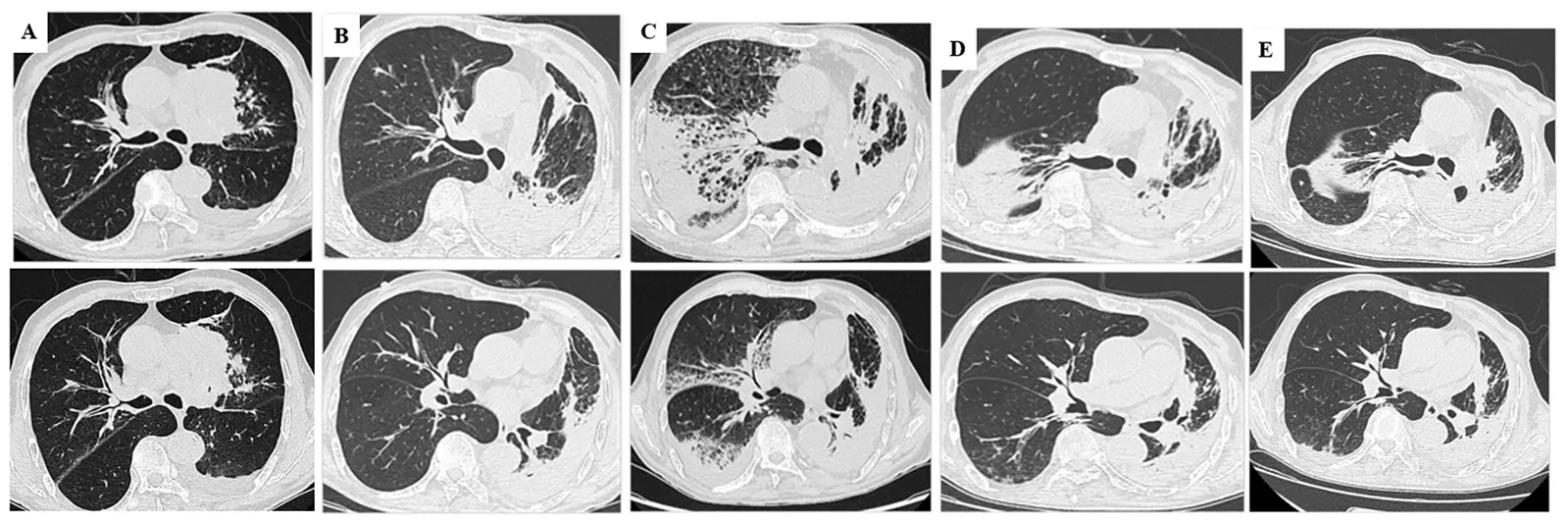
Chest CT of the patient with ES‑SCLC. **(A)** Initial tumor at ES‑SCLC diagnosis. **(B)** After completion of 5 cycles of chemotherapy plus serplulimab, complicated by bacterial pneumonia. **(C)** CIP after serplulimab monotherapy maintenance. **(D)** Follow‑up on the ninth day after hospitalization. **(E)** Follow‑up 2 months after discharge. CIP, checkpoint inhibitor pneumonitis; ES‑SCLC, extensive‑stage small cell lung cancer.

On 30 September 2025, 3 days after the sixth serplulimab dose, the patient developed acute worsening of chest tightness, dyspnea, and hypoxemia that was not relieved by home oxygen therapy; he was eventually admitted to the hospital on 5 October 2025. On arrival, he was bedridden and completely dependent on others for self-care due to severe respiratory distress, corresponding to an ECOG PS of 4. On admission, laboratory evaluation showed a markedly elevated C-reactive protein (CRP) of 153 mg/L, while procalcitonin, neuron-specific enolase (NSE), and carcinoembryonic antigen (CEA) were all within normal ranges. Chest CT demonstrated bilateral multifocal patchy ground-glass opacities and consolidation (**Figure 1C**), with a stable left upper lobe mass compared with post-cycle 5 imaging and no new metastases, ruling out primary SCLC progression. The patient reported a non-productive cough without increased sputum volume or purulent secretions and no recent fever. Serial microbiological testing, including sputum and blood cultures plus multiplex respiratory viral panel, yielded uniformly negative results. Arterial blood gas analysis on 5 L/min nasal oxygen revealed pH 7.50, PO_2_ 46 mmHg, PCO_2_ 33 mmHg, and peripheral oxygen saturation (SpO_2_) 88%–92%, a pattern consistent with hypoxemia-induced hyperventilation leading to respiratory alkalosis. Because hypoxemia remained uncorrected with nasal oxygen, the patient was transitioned to high-flow nasal cannula (HFNC) oxygen therapy (flow rate 45 L/min, FiO_2_ 47%), with SpO_2_ gradually rising to 95%. Given the recent administration of serplulimab and the exclusion of acute exacerbation of COPD and infection, immune-related pneumonitis was the leading diagnosis. Immediately after admission, intravenous methylprednisolone 40 mg twice daily (1.38 mg/kg/day based on actual body weight; height 167 cm, weight 58 kg, Body Mass Index (BMI) 20.8 kg/m^²^) was initiated, along with piperacillin–tazobactam as empiric antimicrobial therapy.

On hospital day 3, the patient’s chest tightness and dyspnea showed no improvement. Despite the escalation of HFNC settings (flow rate 50 L/min, FiO_2_ 55%), SpO_2_ was maintained at only 93%–95%, and the condition was considered to be progressing. At this point, the CIP was classified as grade 4 (life-threatening respiratory compromise) per Common Terminology Criteria for Adverse Events (CTCAE) version 5.0 (15), based on persistent hypoxemia despite escalated HFNC support and progressive respiratory failure in a patient with nearly depleted pulmonary reserve. Invasive mechanical ventilation was recommended given the worsening respiratory failure, but the family declined this intervention. The patient therefore remained on HFNC, with further deterioration in dyspnea over the subsequent 48 h (SpO_2_ < 90%). Methylprednisolone was escalated to 80 mg twice daily, and trimethoprim–sulfamethoxazole was added for *Pneumocystis jirovecii* prophylaxis. An extensive infectious workup (respiratory virus panel, severe acute respiratory syndrome coronavirus 2, 1,3-β-d-glucan, and galactomannan) was negative. Results of admission-day (D1) blood samples became available on hospital day 3: soluble interleukin-2 receptor (sIL-2R) was 2,008.87 U/mL (normal 160–625), and IL-6 was 238.28 pg/mL (normal < 7); meanwhile, CRP had rapidly declined to 29.0 mg/L from 153 mg/L on admission. Given the markedly elevated T-cell activation markers and progressive respiratory failure, intravenous immunoglobulin (IVIG; 20 g/day) was added on the same day as adjunctive immunomodulation to suppress excessive immune activation. The patient showed slight improvement in chest tightness by hospital day 4 after the escalation of corticosteroids, yet the pneumonitis remained severe, with persistent hypoxemic respiratory failure requiring continued HFNC support.

By hospital day 5, CRP had normalized; follow-up (D3-sample) sIL-2R and IL-6 decreased to 1,808.06 U/mL and 104.97 pg/mL, respectively. Bronchoalveolar lavage was deferred due to persistent severe hypoxemia and the high procedural risk associated with HFNC support. Repeated microbiological assessments, including sputum and blood cultures together with targeted serological tests, had yielded persistently negative results; consequently, all empirically administered antimicrobials were tapered progressively and eventually discontinued.

By hospital day 7, the patient showed marked clinical improvement and was transitioned to low-flow oxygen therapy. Serum CRP returned to normal, and D5-sample sIL-2R and IL-6 further declined to 1,139.09 U/mL and 33.9 pg/mL, respectively. Follow-up chest CT on hospital day 10 revealed significant absorption of bilateral pulmonary infiltrates (**Figure 1D**), after which intravenous methylprednisolone was converted to oral prednisone and gradually tapered. Prior to discharge, CRP remained normal on hospital day 13, and both sIL-2R and IL-6 returned to within the reference range on hospital day 15 (D13-sample results). At discharge (hospital day 15, 19 October 2025), the prednisone dose was 20 mg twice daily, with a planned taper over 2 months, along with prophylactic trimethoprim–sulfamethoxazole. Serplulimab was permanently discontinued thereafter. Approximately 2 months after discharge, following CT confirmation of significant resolution of pulmonary inflammation (**Figure 1E**), anlotinib was briefly attempted but self-discontinued after a single dose, and no further anticancer therapy was initiated. No recurrence of CIP was observed during follow-up. A timeline summarizing the clinical course, treatment, and inflammatory markers is shown in **Figure 2**.

**Figure 2:**
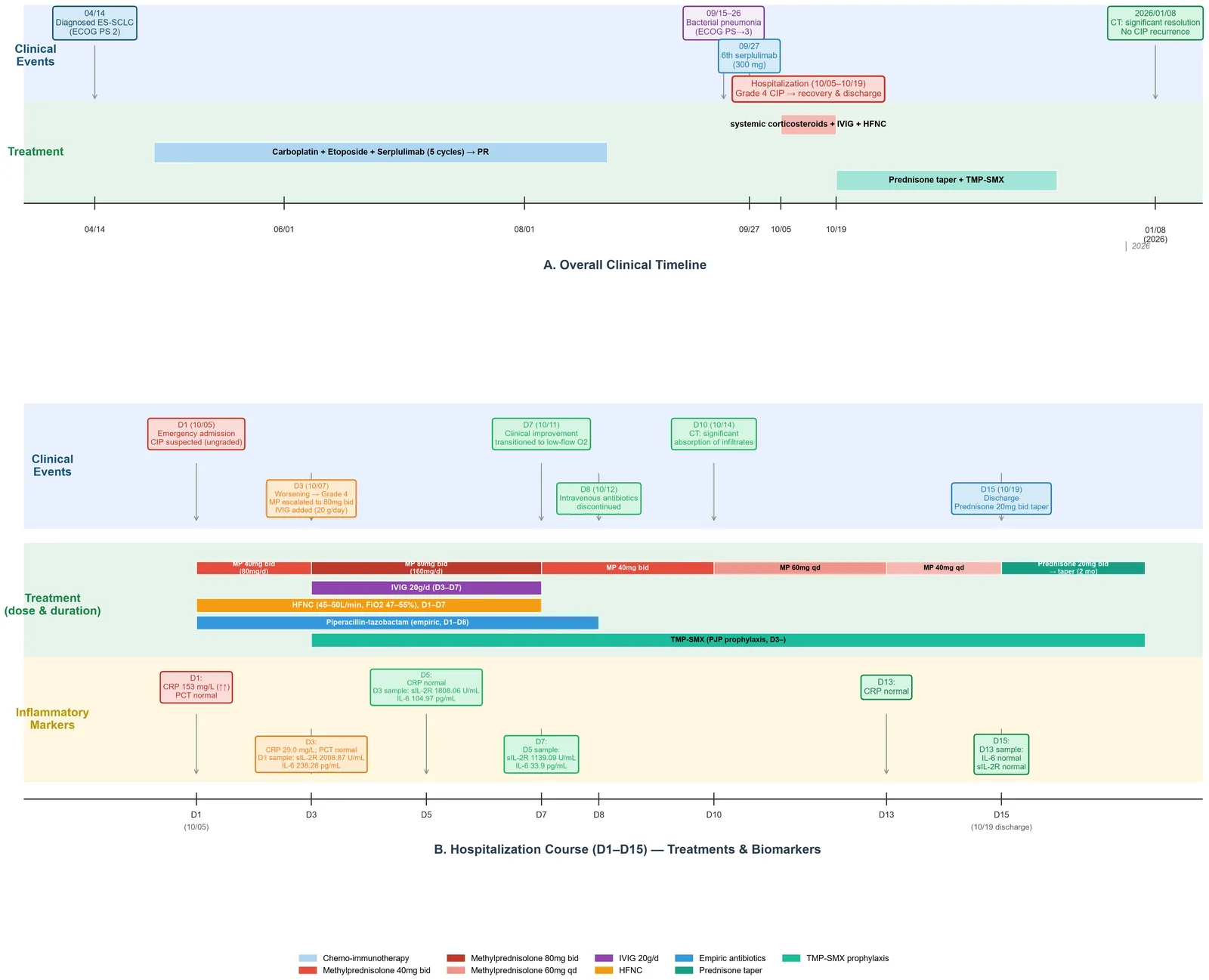
Timeline of the clinical course, treatment, and inflammatory markers. **(A)** Overall clinical timeline from diagnosis of extensive-stage small cell lung cancer (ES-SCLC) through first-line chemoimmunotherapy, bacterial pneumonia, the sixth serplulimab maintenance dose, hospitalization for grade 4 checkpoint inhibitor pneumonitis (CIP), and post-discharge follow-up. **(B)** Detailed hospitalization course (days 1–15) illustrating clinical events, the stepwise corticosteroid regimen (methylprednisolone 40 mg bid → 80 mg bid → 40 mg bid → 60 mg qd → 40 mg qd, followed by oral prednisone taper), adjunctive intravenous immunoglobulin (IVIG), high-flow nasal cannula (HFNC) oxygen therapy, empiric antimicrobial therapy, and dynamic changes in inflammatory markers. C-reactive protein (CRP) and procalcitonin (PCT) were reported on the day of sampling, whereas soluble interleukin-2 receptor (sIL-2R) and interleukin-6 (IL-6) results became available 2 days after sample collection (e.g., day 1 samples reported on day 3). ECOG PS, Eastern Cooperative Oncology Group performance status; TMP-SMX, trimethoprim–sulfamethoxazole; PJP, *Pneumocystis jirovecii* pneumonia.

## Discussion

This case demonstrates that grade 4 CIP can develop during serplulimab maintenance therapy in an elderly patient with ES-SCLC and preexisting GOLD stage IV COPD. The present patient had multiple factors that may increase the risk of CIP, including advanced age, a long-term smoking history, and GOLD stage IV COPD. While COPD has been consistently identified as an independent CIP risk factor in non-SCLC (NSCLC) cohorts (16, 17), and a meta-analysis by Li et al. (14) reported a significantly increased risk (OR = 1.54), SCLC-specific prospective data remain lacking. Deng et al. (18) further validated a risk-scoring model identifying COPD as an independent predictor of all-grade CIP and emphysema/interstitial lung disease as predictors of severe CIP. Preexisting interstitial lung abnormality has also recently been associated with higher CIP risk and worse prognosis in SCLC patients receiving first-line chemoimmunotherapy (19). Whether COPD severity, particularly GOLD stage IV disease, further stratifies CIP risk in SCLC remains unclear, but the present case underscores the need for heightened monitoring in this population.

CIP in SCLC is clinically non-specific, and its radiological patterns, such as ground-glass opacities, consolidation, and organizing pneumonia, overlap with infection, radiation pneumonitis, and COPD exacerbation, making diagnosis particularly challenging in patients with limited pulmonary reserve (20, 21). In the present case, the acute onset of dyspnea and severe hypoxemia without fever or purulent sputum, together with bilateral multifocal ground-glass opacities on CT, favored CIP (21). Acute COPD exacerbation was deemed unlikely given the absence of increased sputum volume or purulent secretions, persistently negative microbiological workup, and hypoxemia with respiratory alkalosis rather than hypercapnia. The combination of markedly elevated CRP with normal procalcitonin further argued against bacterial infection, although this evidence remains supportive rather than definitive. Bronchoscopy was deferred due to the procedural risks of HFNC-dependent respiratory failure, and the diagnosis was therefore established on clinical grounds. Biomarker assessment further corroborated the diagnosis and reflected disease severity. Admission elevations in serum IL-6 and sIL-2R indicated excessive T cell-mediated immune activation, which underlies severe immune-related toxicity (22). Dynamic declines in IL-6 and sIL-2R levels paralleled clinical and radiological improvement, suggesting their potential value in monitoring treatment response. Conventional inflammatory markers such as CRP, Neutrophil-to-Lymphocyte Ratio (NLR), and Lactate Dehydrogenase (LDH) lack sufficient specificity, as similar alterations can occur in infection and tumor progression (23, 24). Lung-specific biomarkers, including KL-6, surfactant protein-D, and CC16 (25), may more precisely reflect alveolar epithelial injury and correlate with CIP severity and treatment response, but were not measured in this case because they are not routinely available at our center. Notably, most biomarker data in CIP derive from NSCLC cohorts (21, 26–28); whether IL-6 and sIL-2R perform similarly in SCLC warrants dedicated investigation.

Permanent discontinuation of serplulimab and timely initiation of systemic corticosteroids remain the cornerstone of CIP management (29–32). The initial methylprednisolone dose of 80 mg/day (1.38 mg/kg/day) was within the guideline-recommended range (1–2 mg/kg/day) for grade 3–4 CIP. However, respiratory deterioration within 48 h prompted escalation to 160 mg/day (2.76 mg/kg/day). This rapid intensification aimed to achieve prompt control and likely contributed to subsequent improvement. We suggest that for patients with preexisting severe COPD and limited pulmonary reserve, higher doses may be warranted if no initial response is observed; timely recognition and dose escalation are critical in this high-risk population. Given persistent hypoxemia despite corticosteroid escalation and markedly elevated sIL-2R/IL-6 indicating T-cell hyperactivation, intravenous immunoglobulin (20 g/day) was added as adjunctive immunomodulation. While IVIG is not a standard or guideline-recommended CIP therapy (30), and high-level evidence supporting its use remains limited, its administration was guided by progressive respiratory failure despite escalated high-dose corticosteroid therapy. Clinical improvement followed IVIG administration, although its independent contribution cannot be definitively separated from the delayed effect of corticosteroid dose escalation. Prophylactic trimethoprim–sulfamethoxazole and empiric antimicrobial therapy were given to reduce infection risk in this immunocompromised patient (30, 33).

Severe CIP is associated with significant mortality across tumor types (34, 35). In SCLC, CIP management strategies are largely extrapolated from NSCLC and pan-tumor guidelines due to limited SCLC-specific evidence; pivotal first-line chemoimmunotherapy trials report that most CIP events are low-grade and responsive to corticosteroid interruption, but severe cases carry substantial mortality (7–9). Outcomes of CIP specifically in SCLC patients with preexisting GOLD stage IV COPD have not been systematically reported. Nevertheless, our patient ultimately achieved clinical recovery after intensified immunosuppressive therapy and had no CIP recurrence during follow-up.

Beyond shared management principles, this case offers three clinically relevant observations for an understudied population. First, it provides real-world safety data in an elderly patient with ES-SCLC and GOLD stage IV COPD, documenting the full clinical course of grade 4 serplulimab-induced CIP and its response to intensified immunosuppression. Second, it illustrates a pragmatic differential diagnostic approach in severe COPD, integrating clinical, laboratory, radiological, and blood gas findings to distinguish CIP from infection and acute COPD exacerbation when bronchoscopy is not feasible. Third, the parallel dynamics of IL-6 and sIL-2R with disease severity suggest their potential as surrogate markers of treatment response in SCLC-associated CIP, although confirmation in larger cohorts is required.

## Conclusion

This case illustrates that severe, life-threatening CIP can develop during serplulimab maintenance therapy in elderly patients with ES-SCLC and preexisting GOLD stage IV COPD. Prompt clinical recognition, timely escalation of corticosteroids combined with adjunctive IVIG, and permanent discontinuation of PD-1 inhibition were pivotal to the successful outcome in this case. For elderly ES-SCLC patients with GOLD stage IV COPD receiving PD-1 inhibitors, close respiratory monitoring and prompt chest CT evaluation for new or worsening respiratory symptoms are warranted to facilitate early CIP detection and prompt initiation of corticosteroid therapy. However, the magnitude of CIP risk conferred by preexisting GOLD stage IV COPD cannot be determined from a single case, and future systematic studies are warranted to establish risk-stratification strategies and optimal management protocols for this vulnerable population.

## Data Availability

The original contributions presented in the study are included in the article/supplementary material. Further inquiries can be directed to the corresponding author.
